# Phthalate Exposures, DNA Methylation and Adiposity in Mexican Children Through Adolescence

**DOI:** 10.3389/fpubh.2019.00162

**Published:** 2019-06-19

**Authors:** Alison Bowman, Karen E. Peterson, Dana C. Dolinoy, John D. Meeker, Brisa N. Sánchez, Adriana Mercado-Garcia, Martha M. Téllez-Rojo, Jaclyn M. Goodrich

**Affiliations:** ^1^Department of Epidemiology, University of Michigan School of Public Health, Ann Arbor, MI, United States; ^2^Department of Nutritional Sciences, University of Michigan School of Public Health, Ann Arbor, MI, United States; ^3^Center for Human Growth and Development, University of Michigan, Ann Arbor, MI, United States; ^4^Department of Environmental Health Sciences, University of Michigan School of Public Health, Ann Arbor, MI, United States; ^5^Department of Epidemiology and Biostatistics, Drexel University Dornsife School of Public Health, Philadelphia, PA, United States; ^6^Center for Research on Nutrition and Health, National Institute of Public Health, Cuernavaca, Mexico

**Keywords:** DNA methylation, epigenetics, environmental exposures, endocrine disrupting chemicals, children, adolescents, anthropometry

## Abstract

Phthalates are a class of endocrine disrupting chemicals with near ubiquitous exposure to populations around the world. Phthalates have been associated with children's adiposity in previous studies, though discrepancies exist across studies that may be due to timing of exposure or outcome assessment and population differences (i.e., genetics, other confounders). DNA methylation, an epigenetic modification involved in gene regulation, may mediate the effects of early life phthalate exposures on health outcomes. This study aims to evaluate the mediating effect of DNA methylation at growth-related genes on the association between phthalate exposure and repeat measures of adiposity (BMI-for-age z-score, waist circumference, and skinfolds thickness) in Mexican children. Urinary phthalate metabolite concentrations were quantified in mothers at each of the three trimesters of pregnancy and in children at the first peri-adolescent study visit. Blood leukocyte DNA methylation at *H19* and *HSD11B2* was quantified during the first peri-adolescent visit, and adiposity was measured at the first visit and again ~3 years later among participants (*n* = 109 boys, 114 girls) from the Early Life Exposure in Mexico to Environmental Toxicants (ELEMENT) project. Associations between phthalates or DNA methylation and repeat outcome measures were assessed separately in boys and girls using generalized estimating equation models including covariates (urinary specific gravity, maternal education, and child's age). Sobel tests were used to assess DNA methylation as a mediator in models adjusting for the same covariates. Associations between phthalates and adiposity varied by phthalate and timing of exposure. Early gestation MBP, MIBP, and MBzP were associated with adiposity among girls. For example, among girls first trimester maternal urine concentrations of MIBP were associated with increases in skinfold thickness, BMI-for-age, and waist circumference (*p* < 0.01). Second trimester and adolescent MBzP were associated with adiposity among boys in opposite directions. In girls, *H19* methylation was positively associated with skinfold thickness. No significant mediation of phthalate exposure on adiposity by DNA methylation of *H19* or *HSD11B2* was observed (Sobel *p* > 0.05). However, the mediation analysis was underpowered to detect small to medium effect sizes, and the role of DNA methylation as a mediator between phthalates and outcomes merits further study.

## Introduction

Endocrine disrupting chemicals (EDCs) represent a class of ubiquitous exposures to humans that disrupt the body's natural hormonal functions and subsequent reproductive and developmental health. Phthalates are a class of EDCs that are used as industrial plasticizers and additives in a wide range of consumer products (1). Phthalates can migrate from the products they are added to into the surrounding environment, which may be food, water, or air that has contacted phthalate-containing plastics (1). Humans can then be exposed to phthalates via ingestion, inhalation, absorption or injection, resulting in high detection frequency of phthalate metabolites in human populations. For example, phthalate metabolites were detected at rates of 79.1–99.3% in a study of U.S. children from California (2) and 95.6–100% among pregnant women from Mexico City (3).

Pregnant women and developing children are particularly susceptible to the endocrine disrupting effects of phthalates and other EDCs (1). Phthalate exposures *in utero* and during childhood have been shown to have lasting health effects including aggressive behavior, learning problems, asthma, allergic symptoms, changes in pubertal timing, and anthropometry (1, 4–6). An effect of particular concern related to phthalate exposure is increased childhood weight status and adiposity. Childhood obesity is highly prevalent in developed nations and has significant potential health implications later in life (7). Previous studies have shown that phthalate exposures, both *in utero* and during adolescence, are associated with measures of weight status and adiposity, with effects varying by phthalate metabolite, timing of exposure and outcome assessment, and sex (6, 8–12).

While mechanisms underlying the various health effects associated with phthalate exposures are not entirely understood, potential mediating pathways include oxidative stress and disruption of metabolic function (13–15). Epigenetic perturbations including DNA methylation are also emerging as potential mechanisms of phthalates' lasting effects (16, 17). The epigenome consists of heritable (mitotically and in some cases meiotically) alterations to the genome that do not affect the genetic sequence but govern the response of cells, tissues, and individuals to their environment (18, 19). There is growing evidence that environmentally-induced epigenetic perturbations, especially during susceptible periods of development such as gestation, can persist throughout life. Exposure to phthalates both *in utero* and in childhood has been associated with DNA methylation at specific genes, including imprinted genes such as *H19*, and repetitive elements (20–22). For example, phthalate exposures both *in utero* and later in development have been shown to correlate with DNA methylation of *H19* and *HSD11B2* in peri-adolescent children (20). *H19* and *HSD11B2* are environmentally responsive genes that serve important roles in regulating growth throughout development. The imprinted *H19* gene is involved in growth and adiposity regulation, especially during development (23). DNA methylation status at this gene measured in 17 year old boys and girls has been associated with greater subcutaneous fat measures (23). Additionally, the *HSD11B2* gene protects cells from the growth-inhibiting and/or pro-apoptotic effects of cortisol, especially during embryonic development (24). *HSD11B2* methylation in placental DNA has been inversely associated with fetal growth (24, 25).

Our prior research in the Early Life Exposure in Mexico to Environmental Toxicants (ELEMENT) project identified associations between phthalate exposure and DNA methylation of *H19* and *HSD11B2* (20) as well as between phthalate exposure and weight status and adiposity at one childhood time point (10, 11). This study aims to extend the evaluation of phthalate exposures and peri-adolescent adiposity to include trimester-specific measures across pregnancy and repeat measures of adiposity in ELEMENT children. Furthermore, we will test whether DNA methylation at *H19* and *HSD11B2* are mediators between phthalate exposures during sensitive developmental periods and measures of peri-adolescent adiposity (BMI-for-age z-score, skinfolds thicknesses, and waist circumference).

## Materials and Methods

### Study Participants

The study population consists of participants from the second and third cohorts of the ELEMENT longitudinal study. Initially, mothers were enrolled during the first trimester of pregnancy or at delivery at maternity hospitals in Mexico City. Mothers recruited in their first trimester (T1) attended follow up visits during their second (T2) and third (T3) trimesters with urine and blood collected at each visit. Children of enrolled mothers attended multiple follow up visits from birth until 5 years of age (*n* = 1,079), with a subset of enrollees returning for additional study visits. At these visits, demographic and dietary data were collected by questionnaire, anthropometric measures were taken, and biospecimen were collected. Complete study methods including exclusion criteria are described elsewhere (26, 27).

This study consists of 250 children who were re-enrolled to attend additional study visits in 2011 and 2012 between the ages of 8 and 14 years during peri-adolescence (referred to here as PA Visit 1), of which 223 children returned betweeen 1.7 and 4.9 (average = 3.4) years later for additional follow-up between the ages of 9 and 17 years (PA Visit 2). Only children attending both study visits are included here. These children were re-recruited from ELEMENT cohorts 2 and 3, prioritizing families with maternal samples from pregnancy available. Blood and urine were collected from the children at both follow up visits.

Mothers received detailed information of study procedures and signed a letter of informed consent at initial recruitment and at follow up in accordance with the Declaration of Helsinki. Children provided assent in written or verbal forms when age-appropriate for follow-up visits. Research protocols were approved by the Ethics and Research Committees of participating institutions in Mexico and the USA including at the University of Michigan.

### Phthalate Metabolite Measurements

Spot urine samples taken at the second morning void were collected from each mother during T1, T2, and T3 as well as from each child at PA visit 1, and stored at −80°C until analysis. Samples were analyzed for nine phthalate metabolites using isotope dilution-liquid chromatography-tandem mass spectrometry (ID LC-MS/MS) according to a validated modification of the Centers for Disease Control and Prevention (CDC) method number 6301.01 as described in detail elsewhere (15, 28). The nine phthalate metabolites measured were monoethyl phthalate (MEP), mono-n-butyl phthalate (MBP), mono-isobutyl phthalate (MiBP), mono(3-carboxypropyl) phthalate (MCPP), monobenzyl phthalate (MBzP), mono(2-ethylhexyl) phthalate (MEHP), mono(2-ethyl-5-hydroxyhexyl) phthalate (MEHHP), mono(2-ethyl-5-oxohexyl) phthalate (MEOHP), and mono(2-ethyl-5-carboxypentyl) phthalate (MECPP). Urinary specific gravity was measured for each sample using a handheld digital refractometer (ATAGO Company Ltd., Tokyo, Japan). Urinary concentrations below the limit of quantitation (LOQ) were assigned a value of LOQ/sqrt (2).

We calculated the molar sum of individual diethylhexyl phthalate (DEHP) metabolites by summing the quotient of metabolite concentrations by their molecular weights (MW) in grams per mole. DEHP molar sum included MEHP (MW 278), MEHHP (MW 294), MEOHP (MW 292), and MECPP (MW 308). The molar sum was then converted to a concentration in μg/L by multiplying by the MW of DEHP.

### DNA Collection, Extraction, and Methylation Analyses

Whole blood samples were collected in PAXgene tubes during the PA Visit 1. DNA was isolated from blood leukocytes using the PAXgene Blood DNA kit (PreAnalytiX, Switzerland). Epitect (Qiagen, Valencia, CA) or EZ DNA Methylation kits (Zymo Research, Irvine, CA) were used according to standard methods to bisulfite convert 0.5–1 μg of genomic DNA, leaving methylated cytosine unchanged and converting unmethylated cytosine to uracil.

The percentage of cells from each sample with methylated DNA was quantified at the H19 paternally imprinted, maternally expressed non-coding RNA transcript and hydroxysteroid (11-beta) dehydrogenase 2, HSD11B2. DNA methylation was measured using pyrosequencing (at 4 CpG sites for H19 and 5 CpG sites for HSD11B2). Additional details on methods including primer sequences and quality control are published elsewhere (20). HSD11B2 and H19 data exhibited batch effects and as such were standardized to controls included on experimental plates as previously described (20). The value of 0% methylation controls on each plate of samples amplified and sequenced together (one laboratory batch) for HSD11B2 was subtracted from the raw DNA methylation values generated for each sample in the same batch, resulting in negative values in some instances.

### Anthropometric Measurements

Children's anthropometry was measured at PA Visits 1 and 2. Waist circumference was measured in duplicate to the nearest 0.1 cm with a non-stretchable tape (QM2000; QuickMedical) (10). Tricep and subscapular skinfold thickness were measured in duplicate to the nearest 0.1 mm with a Lange skinfold caliper (Lange; Beta Technology). Child height was measured in duplicate to the nearest 0.1 cm and weight was measured once. For all outcomes measured in duplicate, if intra-personal variability exceeded the measurement tolerance of +/- 0.5 cm for waist circumference and height or 2.0 mm for skinfold thickness, an additional measurement was taken. Observed values were averaged. To serve as a measure of adiposity, tricep and subscapular skinfold thickness were summed. Additionally, age- and sex-specific BMI z-scores were calculated using the 2007 World Health Organization (WHO) reference growth standard (29).

### Covariates

The number of years of education completed by the mother at enrollment was used as a measure of socioeconomic status. Children's pubertal status was assessed at PA Visits 1 and 2. Pubic hair staging as well as physician observed breast (females only) and genital (males only) development were assessed according to Tanner Staging methodology as we have previously described for this study (10, 15). Initiation of puberty was defined for girls as a pubic hair stage score or breast development stage score >1 and for boys as a pubic hair stage score or genital development stage score >1.

### Statistical Analysis

Data analysis was performed in SAS version 9.4 (SAS Institute, Cary, NC, USA). Individual phthalate metabolite concentrations and molar sum derivatives were natural-log transformed to achieve a normal distribution. All analyses were sex-stratified because phthalate metabolites were previously shown to have sex-specific effects on children's adiposity and on DNA methylation (10, 11, 20). Means, standard deviation (SD), and distributions of phthalate metabolites (geometric means), DNA methylation data, anthropometric outcomes, and maternal education were calculated. To assess whether distributions varied significantly between sexes, Wilcoxon *t*-tests were used for continuous variables and chi-square tests used performed for dichotomous variables.

A series of generalized estimating equation models were run to assess the direct associations between phthalate exposures as well as DNA methylation on adiposity. Separate models were run for phthalate metabolites at each timepoint (T1, T2, T3, PA visit 1) as well as each DNA methylation measure as the exposure with each measure of adiposity (waist circumference, skinfold sum, BMI z-score) as a repeated measure outcome from PA visits 1 and 2. Crude models were run adjusted only for urinary specific gravity at time of urine collection for phthalate metabolites and unadjusted for models of DNA methylation. Adjusted models included specific gravity (for phthalate metabolites), age, and maternal education, with age as a repeated measure for visits 1 and 2. For models with BMI-for-age z-score as the outcome, study visit was included instead of age as a repeated measured. Pubertal status was not included in the final models to avoid potential collider bias since prenatal phthalate exposures have been associated with pubertal timing (30, 31).

Triads of exposure (phthalate metabolite), DNA methylation, and outcome were selected for mediation analysis among boys or girls if both the phthalate and locus-specific DNA methylation were significantly associated with at least one measure of adiposity in a given sex (*p* < 0.1). An alpha value of 10% was used for the selection of variables for mediation analysis so as not to exclude potential associations of interest; an alpha value of 5% was considered statistically significant for all other analyses. Mediation analysis was then performed for that phthalate at each time point with adiposity outcomes and the selected locus. Based on these criteria, MBP, MIBP, MEHP, and MBzP with DNA methylation of *H19* CpG sites 1 and 4 were selected for mediation analysis among girls; MBzP and MEHP and DNA methylation of *HSD11B2* CpG sites 1 and 2 were selected for mediation analysis among boys. Models used in mediation analyses are as follows, with X = exposure (phthalate metabolite), M = mediator (locus-specific DNA methylation), Y = outcome (adiposity measure), γ = intercept, and ε = error:

$$\begin{array}{lll} M & = & \gamma_{1}+\alpha X_{i}+\varepsilon_{1}\\ Y & = & \gamma_{2}+\beta_{total}X_{i}+\varepsilon_{2}\\ Y & = & \gamma_{3}+\beta_{direct}X_{i}+\beta_{mediator}M_{i}+\varepsilon_{3} \end{array}$$

In mediation analyses, total effect was defined as the effect of the exposure on the outcome unadjusted by the mediator (β_*total*_), the direct effect was defined as the effect of the exposure on the outcome adjusted for the mediator (β_*direct*_), and the indirect effect was defined as the product of the effect of the exposure on the mediator and the effect of the mediator on the outcome (α × β_*mediator*_). The statistical significance of a non-zero mediation pathway through the indirect effect was measured using a Sobel test with the formula t = αβ_indirect_/SE, where SE $=\sqrt{\alpha^{2}*\sigma_{\beta \text{indirect}}^{2}+{\beta_{\text{indirect}}}^{2}*\sigma_{\alpha }^{2}}$, ${\sigma_{\alpha }}^{2}$ is the variance of α, and ${\sigma_{\beta \text{indirect}}}^{2}$ is the variance of β_indirect_.

Generalized estimating equations were used for mediation analysis of single exposures (phthalate concentration at a single timepoint), mediators (DNA methylation at a single locus at PA visit 1) and outcomes as repeated measures (skinfold thickness, BMI-for-age z-score, or waist circumference). Mediation analysis models were adjusted for urinary specific gravity, maternal education, and age.

Power calculations were performed to determine the minimum sample size needed to detect a statistically significant Sobel test result with an alpha value of 5% and power of 80%. Various effect size strengths were tested as strong, medium or weak for the effect estimate of the exposure on the mediator and for the mediator on the outcome, adjusted for the exposure. Effect size ranges were determined by the midpoint of tertiles from the absolute value of pooled standardized effect estimates from the mediation models. The sample size calculations were performed in R using the “powerMediation” package.

## Results

### Characteristics of Study Sample

Characteristics of the children at the two study visits in peri-adolescence including adiposity measures and puberty status are found in Table 1. A total of 109 boys and 114 girls were included in the analyses. At both visits, mean BMI-for-age z-score was slightly above zero for boys and girls. DNA methylation levels at PA visit 1 and phthalate concentrations at maternal T1, T2, T3, and PA visit 1 in females and males are presented in Table 2. Percent methylation and urinary phthalate concentrations did not significantly differ between girls and boys, excluding DNA methylation at the second CpG site of *HSD11B2* (higher among girls, *p* = 0.03).

**Table 1:** Characteristics of children at two study visits in peri-adolescence.

	**Male**					**Female**					
	***n***	**Mean or %**	**SD**	**Min**	**Max**	***N***	**Mean or %**	**SD**	**Min**	**Max**	***P*-value**^a^****
**PA VISIT 1**											
Age (years)	109	10.3	1.6	8.1	14.4	114	10.2	1.7	8.1	14.4	0.34
Waist circumference (cm)	109	69.4	9.7	50.1	95.6	114	70.7	10.2	50.5	101.0	0.39
Skinfold thickness sum (mm)	109	25.0	10.7	9.0	64.0	114	29.8	11.3	11.5	56.5	**0.001**
BMI-for-age z-score	109	0.9	1.2	−2.9	4.0	114	0.8	1.3	−2.3	3.7	0.61
Puberty initiation											
Yes	52	49.1				37	32.5				**0.01**^b^
No	54	50.9				77	67.5				
**PA VISIT 2**											
Age (years)	109	13.7	1.8	9.9	17.7	114	13.5	1.8	11.0	17.5	0.52
BMI-for-age z-score	108	0.4	1.3	−3.8	3.4	114	0.6	1.2	−2.2	3.2	0.22
Waist circumference (cm)	109	76.4	10.7	59.9	116.5	114	79.6	10.3	58.5	109.2	**0.02**
Skinfold thickness sum (mm)	109	29.3	14.2	10.5	80	113	39.9	13.9	15.5	78.5	**<0.0001**
Puberty initiation											
Yes	98	92.5				107	95.5				0.34^b^
No	8	7.5				5	4.5				
Maternal education (years)	109	11.2	2.7	3	20	114	10.9	2.8	2	21	0.25

a *T-test for significant difference between boys and girls unless otherwise indicated. Bold values indicates p < 0.05*.

b Chi-square test for significant difference between boys and girls.

**Table 2:** Blood leukocyte DNA methylation (% of methylated cells) at H19 and HSD11B2 at the first peri-adolescent study visit and phthalate metabolite concentrations in urine (μg/L) at multiple time points.

	**Males**			**Females**		
	**n**	**(Geo) Mean^^a^^**	**(Geo) SD**	**n**	**(Geo) Mean^^a^^**	**(Geo) SD**
H19 Methylation						
CpG site 1	107	58.34	7.64	112	59.98	8.85
CpG site 2	107	58.35	3.37	112	58.27	6.13
CpG site 3	107	59.12	3.59	112	59.61	3.90
CpG site 4	107	55.64	7.90	112	57.92	9.18
HSD11B2 Methylation^b^						
CpG site 1	107	−1.48	2.37	113	−1.47	1.88
CpG site 2	107	−0.02	0.93	113	0.25	0.87
CpG site 3	107	−2.17	2.37	112	−2.08	2.10
CpG site 4	107	−0.71	1.60	111	−0.77	1.94
CpG site 5	100	0.25	4.23	103	0.07	4.74
MEP						
T1	85	121.94	3.99	96	137.24	4.20
T2	83	118.05	3.69	96	112.24	3.99
T3	100	114.34	3.77	103	112.80	4.66
PA visit 1	104	65.93	3.50	111	91.78	4.24
MBP						
T1	85	54.75	4.15	96	64.89	3.96
T2	83	40.18	3.74	96	51.76	3.90
T3	100	53.10	3.04	103	57.27	3.70
PA visit 1	104	95.07	2.36	111	109.11	2.95
MIBP						
T1	85	0.88	4.09	96	1.21	3.96
T2	83	0.69	4.10	96	0.91	4.33
T3	100	1.81	2.74	103	2.12	3.01
PA visit 1	104	9.37	2.20	111	11.19	2.40
MCPP						
T1	85	1.11	2.47	96	1.18	2.88
T2	83	1.05	2.55	96	1.07	2.97
T3	100	1.17	2.39	103	1.13	2.81
PA visit 1	104	2.01	2.13	111	2.31	2.85
MBzP						
T1	85	2.55	4.12	96	2.79	3.68
T2	83	2.46	3.75	96	2.23	3.39
T3	100	4.43	2.56	103	4.30	2.76
PA visit 1	104	5.48	2.26	111	5.93	2.53
MECPP						
T1	85	27.68	2.88	96	30.18	2.59
T2	83	28.33	2.43	96	33.60	3.11
T3	100	33.04	2.50	103	31.75	2.97
PA visit 1	104	65.64	2.56	111	62.10	2.32
MEHHP						
T1	85	14.98	3.33	96	16.96	2.92
T2	83	14.95	3.03	96	18.41	3.45
T3	100	19.85	2.88	103	19.38	3.11
PA visit 1	104	47.94	2.68	111	45.17	2.50
MEHP						
T1	85	4.84	2.57	96	5.11	2.91
T2	83	4.39	2.66	96	4.98	2.92
T3	100	5.26	2.72	103	5.42	2.64
PA visit 1	104	6.07	2.91	111	6.00	2.32
MEOHP						
T1	85	8.02	3.27	96	9.13	2.95
T2	83	8.87	3.01	96	11.05	3.42
T3	100	11.93	2.81	103	11.89	3.10
PA visit 1	104	21.16	2.65	111	19.92	2.48
∑DEHP						
T1	85	65.07	2.70	96	71.03	2.63
T2	83	63.42	2.58	96	75.97	3.11
T3	100	78.60	2.51	103	76.69	2.79
PA visit 1	104	157.98	2.59	111	149.46	2.35

a *Means and SD are reported for DNA methylation at H19 and HSD11B2. Geometric means and SD are reported for phthalate metabolites*.

b *These values are first standardized to controls run on each experimental batch, and the standardization procedure results in negative values in some cases*.

### DNA Methylation and Adiposity

Crude and adjusted models assessing the total effect of *H19* and *HSD11B2* methylation on adiposity outcomes are presented in Table 3. In adjusted models, percent methylation at *H19* CpG sites 1 and 4 were positively associated with all three outcomes in females, and this association was statistically significant with skinfold thickness (*p* < 0.05). Percent methylation of *HSD11B2* CpG sites 1 and 2 was inversely associated with all three adiposity outcomes among boys, and the association was near statistically significant for CpG site 1 with skinfold thickness (*p* = 0.05) and CpG site 2 with waist circumference (*p* = 0.08).

**Table 3:** Associations between blood leukocyte DNA methylation and measures of adiposity from a generalized estimating equation relating repeated measures of adiposity outcomes stratified by sex.

	**Skinfold thickness (mm)**		**BMI-for-age z-score**		**Waist circumference (cm)**	
	**Crude model^^a^^**	**Adjusted model**	**Crude model^^a^^**	**Adjusted model**	**Crude model^^a^^**	**Adjusted model**
**FEMALES**						
DNA methylation at *H19*						
CpG site 1	0.15 (−0.11, 0.41)	0.35 (0.07, 0.62)^**^	0.02 (−0.01, 0.05)	0.02 (−0.01, 0.05)	−0.02 (−0.24, 0.2)	0.16 (−0.07, 0.39)
CpG site 2	0.07 (−0.19, 0.33)	0.07 (−0.24, 0.39)	0 (−0.03, 0.02)	0 (−0.03, 0.02)	0.06 (−0.12, 0.24)	0.06 (−0.16, 0.28)
CpG site 3	0.17 (−0.43, 0.76)	0.32 (−0.3, 0.93)	0.01 (−0.05, 0.06)	0.01 (−0.05, 0.06)	0.01 (−0.46, 0.47)	0.14 (−0.33, 0.62)
CpG site 4	0.18 (−0.07, 0.42)	0.36 (0.1, 0.62)^***^	0.02 (0, 0.05)	0.02 (0, 0.05)^*^	0.03 (−0.17, 0.23)	0.2 (−0.02, 0.41)^*^
DNA methylation at *HSD11B2*						
CpG site 1	−0.32 (−1.54, 0.9)	−0.62 (−1.9, 0.67)	−0.04 (−0.17, 0.1)	−0.04 (−0.18, 0.1)	−0.05 (−1.15, 1.05)	−0.34 (−1.46, 0.79)
CpG site 2	0.7 (−1.8, 3.21)	1.68 (−0.96, 4.32)	0.18 (−0.07, 0.42)	0.18 (−0.07, 0.42)	0.32 (−1.76, 2.4)	1.23 (−0.92, 3.37)
CpG site 3	0.08 (−0.89, 1.05)	−0.06 (−1.13, 1)	0.01 (−0.1, 0.12)	0.01 (−0.1, 0.12)	0.26 (−0.64, 1.17)	0.13 (−0.85, 1.1)
CpG site 4	0 (−1.15, 1.14)	0.3 (−0.84, 1.45)	0.07 (−0.03, 0.18)	0.07 (−0.03, 0.18)	0.35 (−0.74, 1.43)	0.63 (−0.43, 1.69)
CpG site 5	−0.04 (−0.55, 0.47)	−0.19 (−0.74, 0.35)	−0.01 (−0.06, 0.04)	−0.01 (−0.06, 0.04)	0 (−0.39, 0.4)	−0.14 (−0.56, 0.28)
**MALES**						
DNA methylation at *H19*						
CpG site 1	0.15 (−0.14, 0.45)	0.19 (−0.1, 0.49)	0.01 (−0.02, 0.04)	0.01 (−0.02, 0.04)	0.04 (−0.21, 0.28)	0.11 (−0.13, 0.34)
CpG site 2	0.06 (−0.64, 0.76)	0.03 (−0.69, 0.75)	0.02 (−0.06, 0.1)	0.02 (−0.05, 0.1)	0.12 (−0.43, 0.67)	0.07 (−0.5, 0.64)
CpG site 3	0.4 (−0.26, 1.05)	0.46 (−0.22, 1.15)	0.04 (−0.03, 0.11)	0.04 (−0.03, 0.11)	0.12 (−0.38, 0.63)	0.25 (−0.28, 0.77)
CpG site 4	0.11 (−0.16, 0.39)	0.16 (−0.12, 0.43)	0.01 (−0.02, 0.03)	0 (−0.02, 0.03)	0.02 (−0.21, 0.25)	0.09 (−0.12, 0.3)
DNA methylation at *HSD11B2*						
CpG site 1	−0.93 (−1.96, 0.1)^*^	−1.04 (−2.1, 0.02)^*^	−0.05 (−0.16, 0.06)	−0.05 (−0.17, 0.06)	−0.5 (−1.38, 0.38)	−0.69 (−1.61, 0.23)
CpG site 2	−2.01 (−4.55, 0.53)	−2.07 (−4.68, 0.53)	−0.2 (−0.47, 0.07)	−0.21 (−0.48, 0.06)	−1.7 (−3.68, 0.28)^*^	−1.84 (−3.87, 0.19)^*^
CpG site 3	−0.4 (−1.33, 0.53)	−0.41 (−1.37, 0.55)	0.01 (−0.09, 0.11)	0.01 (−0.08, 0.11)	−0.28 (−1.06, 0.49)	−0.3 (−1.1, 0.49)
CpG site 4	0.43 (−0.77, 1.63)	0.49 (−0.79, 1.77)	0.05 (−0.1, 0.2)	0.05 (−0.1, 0.19)	0.47 (−0.64, 1.59)	0.56 (−0.63, 1.75)
CpG site 5	−0.09 (−0.64, 0.46)	−0.13 (−0.67, 0.42)	0 (−0.06, 0.05)	0 (−0.06, 0.05)	−0.03 (−0.5, 0.44)	−0.09 (−0.54, 0.35)

* p < 0.10,

** *p **<0.05***,

*** *p **<0.01***.

a *Crude models are unadjusted and adjusted models include maternal education and age. Effect estimate (95% confidence intervals) are displayed*.

### Prenatal Phthalate Exposure Biomarkers and Adiposity

We ran crude and adjusted models to assess the total effect of trimester-specific phthalate exposure on adiposity outcomes for females (Table 4) and males (Table 5). Three phthalate metabolites at earlier gestational periods were associated with at least one measure of girls' adiposity in adjusted models at the 95% confidence level: MBP (T1), MIBP (T1), and MBzP (T2; Table 4). Positive associations were observed between T1 MBP and MIBP, both metabolites of dibutyl phthalate (DBP), and all outcomes (for MBP, *p*-value = 0.03 and 0.06 for adjusted models of BMI and waist circumference; for MIBP, *p* = 0.0005, 0.0008, and 0.0019 for skinfold thickness, BMI, and waist circumference). MBzP from T2 and T3 were inversely associated with all three adiposity outcomes among girls, and T2 MBzP was significantly associated with decreased skinfold thickness (*p* = 0.03). MEHP was also considered for mediation analysis as T1 MEHP was inversely associated with waist circumference (*p* = 0.08). Among boys, after covariate adjustment T2 MBzP was associated with increased BMI and waist circumference (*p* = 0.04 and 0.02; Table 5). MEHP was considered in mediation analysis as T1 MEHP was positively associated with skinfold thickness (*p* = 0.08) and BMI (0.05).

**Table 4:** Associations between natural log–transformed phthalate concentrations (μg/L) and measures of adiposity from a generalized estimating equation relating repeated measures of adiposity outcomes among females.

	**Skinfold thickness (mm)**		**BMI–for–age z–score**		**Waist circumference (cm)**	
	**Crude model^**a**^**	**Adjusted model**	**Crude model^**a**^**	**Adjusted model**	**Crude model^**a**^**	**Adjusted model**
MEP						
T1	0.80 (−0.82, 2.42)	1.27 (−0.52, 3.06)	0.06 (−0.12, 0.23)	0.06 (−0.12, 0.24)	0.24 (−1.26, 1.74)	0.66 (−0.95, 2.27)
T2	0.69 (−1.64, 3.02)	0.54 (−1.82, 2.90)	0.00 (−0.21, 0.22)	0.02 (−0.20, 0.25)	0.29 (−1.72, 2.30)	0.07 (−1.86, 2.00)
T3	1.16 (−0.79, 3.12)	1.24 (−0.93, 3.42)	0.01 (−0.21, 0.23)	0.01 (−0.21, 0.23)	0.13 (−1.61, 1.87)	0.21 (−1.63, 2.05)
PA visit 1	0.49 (−1.01, 1.99)	0.13 (−1.45, 1.72)	0.05 (−0.1, 0.20)	0.05 (−0.10, 0.20)	0.37 (−0.95, 1.68)	0.03 (−1.18, 1.24)
MBP						
T1	1.20 (−0.79, 3.18)	1.88 (−0.42, 4.18)	0.24 (0.03, 0.46)^**^	0.25 (0.03, 0.46)^**^	1.09 (−0.52, 2.71)	1.72 (−0.09, 3.53)^*^
T2	−1.13 (−3.44, 1.19)	−1.45 (−3.67, 0.77)	−0.12 (−0.32, 0.08)	−0.12 (−0.32, 0.08)	−0.91 (−2.71, 0.89)	−1.20 (−2.94, 0.53)
T3	0.23 (−1.99, 2.45)	0.05 (−2.23, 2.32)	−0.06 (−0.25, 0.14)	−0.06 (−0.26, 0.14)	−0.20 (−2.02, 1.61)	−0.36 (−2.09, 1.36)
PA visit 1	−1.54 (−3.90, 0.82)	−2.28 (−4.86, 0.29)^*^	−0.21 (−0.46, 0.03)^*^	−0.21 (−0.46, 0.03)^*^	−0.94 (−2.93, 1.04)	−1.63 (−3.65, 0.40)
MIBP						
T1	2.16 (0.31, 4.01)^**^	3.41 (1.50, 5.31)^***^	0.28 (0.11, 0.45)^***^	0.28 (0.12, 0.45)^***^	1.19 (−0.29, 2.66)	2.33 (0.86, 3.8)^***^
T2	0.82 (−1.44, 3.09)	1.46 (−0.76, 3.68)	0.03 (−0.19, 0.25)	0.03 (−0.19, 0.24)	−0.06 (−2.29, 2.18)	0.55 (−1.61, 2.72)
T3	1.21 (−1.96, 4.37)	2.17 (−1.23, 5.56)	0.11 (−0.21, 0.42)	0.10 (−0.20, 0.40)	0.64 (−1.92, 3.21)	1.55 (−1.14, 4.23)
PA visit 1	−1.15 (−3.93, 1.62)	−0.38 (−3.23, 2.47)	−0.10 (−0.4, 0.20)	−0.10 (−0.39, 0.20)	−1.85 (−3.97, 0.28)^*^	−1.15 (−3.29, 0.99)
MCPP						
T1	0.30 (−1.93, 2.53)	0.97 (−1.64, 3.58)	0.18 (−0.08, 0.44)	0.18 (−0.08, 0.45)	0.39 (−1.47, 2.25)	1.01 (−1.03, 3.04)
T2	−1.59 (−4.55, 1.37)	−1.49 (−4.36, 1.38)	−0.16 (−0.42, 0.11)	−0.15 (−0.40, 0.11)	−1.50 (−3.85, 0.86)	−1.45 (−3.67, 0.78)
T3	−0.96 (−3.74, 1.83)	−1.08 (−4.03, 1.87)	−0.11 (−0.36, 0.15)	−0.11 (−0.36, 0.14)	−0.50 (−2.68, 1.68)	−0.60 (−2.84, 1.63)
PA visit 1	−0.96 (−3.48, 1.55)	−1.30 (−4.15, 1.54)	−0.19 (−0.45, 0.07)	−0.19 (−0.44, 0.07)	−0.93 (−2.92, 1.06)	−1.25 (−3.41, 0.90)
MBzP						
T1	−0.51 (−2.38, 1.36)	−0.10 (−2.21, 2.00)	0.04 (−0.17, 0.25)	0.04 (−0.18, 0.25)	−0.37 (−1.86, 1.12)	0.03 (−1.70, 1.76)
T2	**−2.31 (−4.56**, −0.06)^**^	**−2.53 (−4.78**, −0.28)^**^	−0.21 (−0.45, 0.03)^*^	−0.22 (−0.46, 0.03)^*^	−1.82 (−3.89, 0.24)^*^	−2.01 (−4.07, 0.05)^*^
T3	−0.83 (−3.25, 1.59)	−1.20 (−3.81, 1.42)	−0.22 (−0.45, 0.01)^*^	−0.23 (−0.46, 0.01)^*^	−1.00 (−3.07, 1.07)	−1.32 (−3.37, 0.73)
PA visit 1	0.52 (−2.44, 3.48)	0.61 (−2.67, 3.88)	0.11 (−0.21, 0.42)	0.10 (−0.23, 0.43)	−0.40 (−2.92, 2.13)	−0.31 (−3.11, 2.49)
MECPP						
T1	−0.09 (−2.93, 2.76)	0.68 (−2.35, 3.70)	0.13 (−0.16, 0.41)	0.13 (−0.16, 0.41)	−0.25 (−2.47, 1.97)	0.48 (−1.90, 2.87)
T2	−0.84 (−3.84, 2.15)	−1.24 (−4.61, 2.13)	−0.06 (−0.40, 0.27)	−0.08 (−0.42, 0.26)	0.27 (−2.53, 3.07)	−0.05 (−3.11, 3.02)
T3	−0.04 (−2.87, 2.78)	−0.72 (−3.63, 2.19)	−0.01 (−0.25, 0.23)	−0.02 (−0.26, 0.21)	0.64 (−1.84, 3.11)	0.07 (−2.29, 2.43)
PA visit 1	−0.13 (−3.22, 2.95)	0.74 (−2.73, 4.21)	0.11 (−0.23, 0.45)	0.10 (−0.25, 0.45)	−0.40 (−3.1, 2.31)	0.43 (−2.47, 3.33)
MEHHP						
T1	0.07 (−2.34, 2.47)	0.89 (−1.72, 3.49)	0.13 (−0.11, 0.38)	0.13 (−0.12, 0.38)	−0.14 (−1.98, 1.70)	0.64 (−1.41, 2.68)
T2	−1.02 (−3.61, 1.57)	−1.35 (−4.19, 1.49)	−0.11 (−0.40, 0.19)	−0.12 (−0.42, 0.17)	−0.39 (−2.88, 2.11)	−0.63 (−3.33, 2.07)
T3	−0.07 (−2.67, 2.53)	−0.56 (−3.22, 2.10)	−0.03 (−0.25, 0.19)	−0.03 (−0.25, 0.19)	0.39 (−1.80, 2.58)	−0.03 (−2.16, 2.09)
PA visit 1	0.10 (−2.87, 3.06)	1.12 (−2.29, 4.52)	0.12 (−0.22, 0.45)	0.11 (−0.23, 0.45)	−0.52 (−3.06, 2.03)	0.44 (−2.37, 3.26)
MEHP						
T1	−2.10 (−4.62, 0.42)	−1.95 (−4.46, 0.56)	−0.13 (−0.37, 0.12)	−0.13 (−0.38, 0.12)	−1.72 (−3.53, 0.09)^*^	−1.57 (−3.33, 0.20)^*^
T2	−1.48 (−4.23, 1.27)	−2.07 (−4.97, 0.83)	−0.16 (−0.47, 0.14)	−0.19 (−0.50, 0.13)	−1.24 (−3.89, 1.40)	−1.72 (−4.54, 1.10)
T3	−1.09 (−4.20, 2.03)	−1.35 (−4.39, 1.7)	−0.11 (−0.36, 0.14)	−0.11 (−0.36, 0.14)	−0.16 (−2.55, 2.23)	−0.40 (−2.60, 1.79)
PA visit 1	−0.96 (−4.12, 2.20)	−0.77 (−4.14, 2.61)	−0.03 (−0.35, 0.29)	−0.03 (−0.35, 0.29)	−0.80 (-3.48, 1.88)	−0.6 (−3.42, 2.21)
MEOHP						
T1	−0.20 (−2.61, 2.20)	0.46 (−2.13, 3.06)	0.09 (−0.15, 0.34)	0.09 (−0.16, 0.34)	−0.40 (−2.21, 1.41)	0.24 (−1.78, 2.26)
T2	−1.20 (−3.84, 1.43)	−1.73 (−4.65, 1.19)	−0.13 (−0.44, 0.17)	−0.15 (−0.45, 0.15)	−0.56 (−3.14, 2.02)	−0.99 (−3.81, 1.83)
T3	0.01 (−2.64, 2.67)	−0.56 (−3.32, 2.20)	−0.03 (−0.24, 0.18)	−0.04 (−0.24, 0.17)	0.38 (−1.75, 2.51)	−0.11 (−2.19, 1.97)
PA visit 1	−0.44 (−3.43, 2.54)	0.56 (−2.87, 3.99)	0.05 (−0.28, 0.38)	0.04 (−0.30, 0.39)	−0.97 (−3.54, 1.59)	−0.03 (−2.87, 2.82)
∑DEHP						
T1	−0.66 (−3.45, 2.12)	0.06 (−2.82, 2.95)	0.08 (−0.20, 0.36)	0.08 (−0.20, 0.36)	−0.61 (−2.77, 1.54)	0.08 (−2.20, 2.36)
T2	−1.02 (−3.99, 1.95)	−1.44 (−4.77, 1.89)	−0.09 (−0.43, 0.25)	−0.11 (−0.45, 0.23)	−0.13 (−2.98, 2.71)	−0.46 (−3.57, 2.66)
T3	−0.07 (−3.08, 2.94)	−0.75 (−3.89, 2.39)	−0.02 (−0.27, 0.23)	−0.03 (−0.28, 0.21)	0.69 (−1.72, 3.11)	0.11 (−2.28, 2.50)
PA visit 1	−0.07 (−3.18, 3.04)	0.87 (−2.65, 4.39)	0.11 (−0.23, 0.46)	0.10 (−0.25, 0.46)	−0.47 (−3.18, 2.24)	0.42 (−2.51, 3.36)

* p < 0.10,

** **p<0.05**,

*** ***p < 0.01***.

a *Crude models are adjusted for specific gravity. Adjusted models include specific gravity, maternal education, and age. Effect estimates (95% CI) are displayed*.

**Table 5:** Associations between natural log-transformed phthalate concentrations (μg/L) and measures of adiposity from a generalized estimating equation relating repeated measures of adiposity outcomes among males.

	**Skinfold thickness (mm)**		**BMI-for-age z-score**		**Waist circumference (cm)**	
	**Crude model^**a**^**	**Adjusted model**	**Crude model^**a**^**	**Adjusted model**	**Crude model^**a**^**	**Adjusted model**
MEP						
T1	0.05 (−1.84, 1.95)	−0.08 (−1.95, 1.80)	0.01 (−0.15, 0.18)	0.01 (−0.15, 0.18)	−0.25 (−1.65, 1.15)	−0.50 (−1.88, 0.88)
T2	−0.10 (−2.79, 2.59)	−0.30 (−2.99, 2.39)	−0.03 (−0.23, 0.17)	−0.03 (−0.24, 0.17)	−0.08 (−1.83, 1.68)	−0.47 (−2.15, 1.21)
T3	−0.16 (−2.55, 2.22)	−0.37 (−2.75, 2.01)	−0.06 (−0.24, 0.12)	−0.06 (−0.25, 0.12)	−0.21 (−1.84, 1.42)	−0.62 (−2.17, 0.92)
PA visit 1	−1.11 (−2.78, 0.56)	−1.30 (−2.97, 0.36)	−0.07 (−0.23, 0.09)	−0.08 (−0.24, 0.08)	−0.47 (−1.83, 0.89)	−0.85 (−2.18, 0.47)
MBP						
T1	0.33 (−1.38, 2.03)	0.46 (−1.38, 2.31)	0.04 (−0.15, 0.23)	0.04 (−0.15, 0.24)	0.03 (−1.4, 1.46)	0.04 (−1.49, 1.58)
T2	−0.89 (−3.61, 1.83)	−1.24 (−4.14, 1.66)	0.00 (−0.27, 0.27)	−0.01 (−0.29, 0.27)	−0.10 (−2.07, 1.87)	−0.71 (−2.76, 1.35)
T3	0.03 (−2.25, 2.30)	−0.10 (−2.63, 2.42)	0.02 (−0.21, 0.24)	0.00 (−0.23, 0.23)	0.18 (−1.52, 1.89)	−0.16 (−1.97, 1.65)
PA visit 1	0.50 (−2.38, 3.38)	0.86 (−2.1, 3.82)	0.03 (−0.25, 0.31)	0.03 (−0.25, 0.31)	−0.39 (−2.51, 1.73)	0.19 (−1.98, 2.35)
MIBP						
T1	−0.29 (−2.10, 1.52)	0.27 (−1.53, 2.08)	0.02 (−0.16, 0.21)	0.02 (−0.16, 0.20)	−0.53 (−2.00, 0.94)	0.18 (−1.37, 1.72)
T2	0.16 (−2.61, 2.93)	0.41 (−2.39, 3.22)	0.11 (−0.15, 0.36)	0.10 (−0.14, 0.35)	0.53 (−1.46, 2.53)	0.84 (−1.21, 2.88)
T3	−1.47 (−3.77, 0.84)	−1.39 (−3.98, 1.19)	−0.07 (−0.29, 0.15)	−0.10 (−0.33, 0.13)	−1.03 (−2.89, 0.84)	−1.01 (−2.96, 0.94)
PA visit 1	0.25 (−2.74, 3.25)	−0.11 (−3.12, 2.91)	0.00 (−0.30, 0.29)	−0.01 (−0.31, 0.28)	0.80 (−1.39, 3.00)	0.12 (−1.95, 2.18)
MCPP						
T1	1.88 (−1.22, 4.98)	2.29 (−0.95, 5.53)	0.25 (−0.06, 0.57)	0.27 (−0.05, 0.59)	1.69 (−0.71, 4.10)	1.96 (−0.39, 4.30)
T2	1.26 (−2.19, 4.71)	0.90 (−2.68, 4.49)	0.12 (−0.21, 0.45)	0.12 (−0.23, 0.46)	1.44 (−1.16, 4.04)	0.71 (−1.98, 3.41)
T3	0.21 (−2.90, 3.32)	0.33 (−3.1, 3.75)	0.03 (−0.28, 0.33)	0.01 (−0.30, 0.32)	0.14 (−2.12, 2.40)	0.19 (−2.25, 2.63)
PA visit 1	−2.34 (−5.22, 0.55)	−1.81 (−4.73, 1.11)	−0.21 (−0.49, 0.07)	−0.23 (−0.51, 0.06)	**−2.59 (−4.78**, −0.41)**	−1.72 (−3.82, 0.37)
MBzP						
T1	1.48 (−0.66, 3.61)	1.91 (−0.30, 4.12)*	0.18 (−0.01, 0.38)*	0.19 (0.00, 0.39)*	1.13 (−0.49, 2.75)	1.55 (−0.13, 3.22)*
T2	2.07 (−0.30, 4.43)*	2.00 (−0.43, 4.43)	0.25 (0.01, 0.49)**	0.25 (0.01, 0.49)**	2.28 (0.48, 4.08)**	2.11 (0.27, 3.95)**
T3	−0.19 (−2.67, 2.29)	−0.49 (−2.93, 1.94)	−0.12 (−0.34, 0.09)	−0.12 (−0.33, 0.09)	0.15 (−1.97, 2.27)	−0.35 (−2.24, 1.55)
PA visit 1	**−2.50 (−4.83**, −0.16)**	**−2.43 (−4.69**, −0.17)**	−0.16 (−0.40, 0.08)	−0.17 (−0.41, 0.07)	**−1.99 (−3.92**, −0.05)**	**−1.91 (−3.64**, −0.19)**
MECPP						
T1	2.08 (−0.96, 5.13)	2.28 (−0.76, 5.31)	0.20 (−0.11, 0.52)	0.20 (−0.11, 0.52)	1.23 (−1.30, 3.75)	1.42 (−1.14, 3.98)
T2	0.70 (−3.23, 4.62)	0.54 (−3.29, 4.37)	0.24 (−0.16, 0.65)	0.25 (−0.16, 0.65)	1.89 (−1.19, 4.96)	1.73 (−1.27, 4.73)
T3	−0.13 (−2.84, 2.58)	−0.07 (−2.75, 2.61)	−0.05 (−0.35, 0.25)	−0.03 (−0.33, 0.27)	−0.69 (−3.1, 1.71)	−0.42 (−2.69, 1.85)
PA visit 1	−0.76 (−2.81, 1.29)	−0.75 (−2.91, 1.42)	−0.07 (−0.28, 0.14)	−0.10 (−0.31, 0.12)	−0.44 (−2.01, 1.13)	−0.49 (−2.07, 1.09)
MEHHP						
T1	1.56 (−0.85, 3.98)	1.88 (−0.56, 4.32)	0.19 (−0.05, 0.43)	0.20 (−0.04, 0.44)	0.97 (−0.98, 2.92)	1.30 (−0.7, 3.29)
T2	0.65 (−2.28, 3.58)	0.41 (−2.54, 3.37)	0.18 (−0.12, 0.49)	0.19 (−0.12, 0.49)	1.61 (−0.54, 3.76)	1.30 (−0.93, 3.53)
T3	−0.47 (−2.79, 1.85)	−0.45 (−2.74, 1.83)	−0.01 (−0.26, 0.25)	0.01 (−0.25, 0.26)	−0.59 (−2.58, 1.40)	−0.44 (−2.29, 1.41)
PA visit 1	−0.56 (−2.4, 1.29)	−0.55 (−2.49, 1.38)	−0.05 (−0.24, 0.14)	−0.07 (−0.26, 0.12)	−0.46 (−1.83, 0.91)	−0.56 (−1.88, 0.76)
MEHP						
T1	2.40 (−0.61, 5.41)	2.67 (−0.37, 5.71)*	0.28 (0.00, 0.56)*	0.28 (0.00, 0.57)*	1.58 (−0.89, 4.05)	1.86 (−0.61, 4.33)
T2	1.06 (−1.90, 4.02)	0.63 (−2.42, 3.67)	0.24 (−0.08, 0.55)	0.23 (−0.08, 0.55)	2.10 (−0.19, 4.39)*	1.41 (−0.91, 3.74)
T3	−0.33 (−3.28, 2.62)	−0.63 (−3.63, 2.37)	0.08 (−0.19, 0.35)	0.09 (−0.18, 0.37)	0.09 (−2.15, 2.34)	−0.34 (−2.57, 1.89)
PA visit 1	−1.40 (−3.32, 0.52)	−1.90 (−3.91, 0.11)*	−0.13 (−0.32, 0.06)	−0.15 (−0.36, 0.05)	−0.47 (−1.99, 1.06)	−1.33 (−2.90, 0.24)
MEOHP						
T1	1.59 (−0.86, 4.03)	1.88 (−0.59, 4.35)	0.20 (−0.04, 0.44)	0.20 (−0.04, 0.44)	1.07 (−0.87, 3.02)	1.37 (−0.61, 3.36)
T2	0.79 (−2.26, 3.83)	0.48 (−2.56, 3.52)	0.19 (−0.12, 0.50)	0.19 (−0.12, 0.5)	1.80 (−0.45, 4.04)	1.39 (−0.89, 3.68)
T3	−0.69 (−3.09, 1.70)	−0.76 (−3.10, 1.58)	−0.06 (−0.3, 0.19)	−0.04 (−0.29, 0.20)	−0.75 (−2.79, 1.29)	−0.74 (−2.59, 1.12)
PA visit 1	−0.75 (−2.57, 1.08)	−0.75 (−2.68, 1.18)	−0.08 (−0.27, 0.1)	−0.11 (−0.29, 0.08)	−0.56 (−2.00, 0.88)	−0.65 (−2.03, 0.73)
∑DEHP						
T1	2.28 (−0.95, 5.5)	2.58 (−0.66, 5.83)	0.26 (−0.05, 0.57)	0.26 (−0.05, 0.57)	1.39 (−1.19, 3.97)	1.71 (−0.94, 4.36)
T2	0.80 (−2.64, 4.25)	0.56 (−2.87, 4.00)	0.24 (−0.13, 0.60)	0.24 (−0.13, 0.61)	1.94 (−0.73, 4.60)	1.63 (−1.06, 4.32)
T3	−0.43 (−3.15, 2.29)	−0.41 (−3.09, 2.27)	−0.03 (−0.32, 0.26)	−0.01 (−0.30, 0.29)	−0.75 (−3.12, 1.61)	−0.56 (−2.77, 1.64)
PA visit 1	−0.72 (−2.67, 1.23)	−0.76 (−2.79, 1.28)	−0.07 (−0.27, 0.13)	−0.09 (−0.30, 0.11)	−0.44 (−1.92, 1.04)	−0.59 (−2.05, 0.86)

* *p <0.10*,

** ***p < 0.05***.

a *Crude models are adjusted for specific gravity. Adjusted models include specific gravity, maternal education, and age. Effect estimates (95% CI) are displayed*.

### Peri-Adolescent Phthalate Exposure Biomarkers and Adiposity

We examined associations between children's urinary phthalate concentrations at visit 1 with adiposity measures from visits 1 and 2. Significant associations between childhood phthalates and outcomes were only observed among boys. MBzP was inversely associated with skinfold thickness (*p* = 0.04) and waist circumference (*p* = 0.03) among boys in adjusted models.

### Mediation Analysis

Results (total, direct, and indirect effect estimates) from mediation analysis are presented in Table 6 for girls and Table 7 for boys; for additional estimates from the mediation models see Supplemental Tables 1, 2. For both boys and girls, the Sobel tests revealed no statistically significant mediation effects for DNA methylation at the selected loci in the association between the phthalates and the outcome measures at the 95% confidence level. The final sample size for analysis in this study was 114 for girls and 109 for boys, although missing data for specific exposures or mediators decreased some model sample sizes further. Table 8 displays minimum sample sizes needed to detect a significant mediation pathway via the Sobel test given specified effect sizes for standardized exposures, standardized mediators, and skinfold thickness as the outcome. Given the required sample sizes, this study has enough observations to detect significant mediation pathways via the Sobel test with strong effect sizes, but lacked the statistical power to detect significant associations with small or medium effect sizes. Thus, while there were no statistically significant Sobel tests, there were several notable findings (Sobel test *p* ≤ 0.10) which we will discuss as areas for future study.

**Table 6:** Mediation analysis among females.

	**Skinfold thickness (mm)**			**BMI for age z-score**			**Waist circumference (cm)**		
**Exposure**	**Total effect**	**Direct effect**	**Indirect effect (Sobel *p*-value)**	**Total effect**	**Direct effect**	**Indirect effect (Sobel *p*-value)**	**Total effect**	**Direct effect**	**Indirect effect (Sobel *p*-value)**
***H19*** **CpG #4 AS MEDIATOR**									
MBP									
T1	1.88 (−0.42, 4.18)	1.46 (−0.92, 3.83)	0.41 (0.16)	0.25 (0.03, 0.46)^**^	0.22 (0, 0.43)^*^	0.02 (0.27)	1.72 (−0.09, 3.53)^*^	1.42 (−0.44, 3.27)	0.24 (0.24)
T2	−1.45 (−3.67, 0.77)	−1 (−3.32, 1.33)	−0.37 (0.27)	−0.12 (−0.32, 0.08)	−0.08 (−0.28, 0.12)	−0.02 (0.33)	−1.2 (−2.94, 0.53)	−0.9 (−2.63, 0.82)	−0.2 (0.34)
T3	0.05 (−2.23, 2.32)	0.24 (−2.22, 2.71)	−0.17 (0.62)	−0.06 (−0.26, 0.14)	−0.04 (−0.24, 0.16)	−0.01 (0.62)	−0.36 (−2.09, 1.36)	−0.21 (−1.94, 1.53)	−0.09 (0.63)
PA visit 1	−2.28 (−4.86, 0.29)^*^	−2.55 (−5.15, 0.05)^*^	0.37 (0.28)	−0.21 (−0.46, 0.03)^*^	−0.23 (−0.48, 0.02)^*^	0.02 (0.32)	−1.63 (−3.65, 0.4)	−1.78 (−3.84, 0.28)^*^	0.18 (0.34)
MiBP									
T1	3.41 (1.5, 5.31)^***^	2.9 (0.76, 5.03)^**^	0.47 (0.16)	0.28 (0.12, 0.45)^***^	0.25 (0.07, 0.43)^**^	0.03 (0.35)	2.33 (0.86, 3.8)^***^	2.05 (0.38, 3.73)^**^	0.27 (0.3)
T2	1.46 (−0.76, 3.68)	0.81 (−1.69, 3.31)	0.63 (0.11)	0.03 (−0.19, 0.24)	−0.02 (−0.26, 0.22)	0.05 (0.18)	0.55 (−1.61, 2.72)	0.26 (−2.17, 2.69)	0.36 (0.22)
T3	2.17 (−1.23, 5.56)	1.85 (−1.7, 5.4)	0.37 (0.38)	0.1 (−0.2, 0.4)	0.08 (−0.23, 0.39)	0.02 (0.41)	1.55 (−1.14, 4.23)	1.5 (−1.25, 4.25)	0.2 (0.42)
PA visit 1	−0.38 (−3.23, 2.47)	−0.29 (−3.22, 2.64)	0.22 (0.55)	−0.1 (−0.39, 0.2)	−0.09 (−0.39, 0.22)	0.01 (0.57)	−1.15 (−3.29, 0.99)	−1.17 (−3.36, 1.03)	0.11 (0.57)
MBzP									
T1	−0.1 (−2.21, 2.00)	−0.71 (−2.83, 1.41)	0.5 (0.16)	0.04 (−0.18, 0.25)	0 (−0.22, 0.21)	0.03 (0.23)	0.03 (−1.7, 1.76)	−0.3 (−2.06, 1.47)	0.3 (0.22)
T2	**−2.53 (−4.78**, −0.28)^**^	**−2.72 (−4.74**, −0.69)^**^	0.07 (0.85)	−0.22 (−0.46, 0.03)^*^	−0.23 (−0.46, 0)^*^	0.00 (0.85)	−2.01 (−4.07, 0.05)^*^	**−2.08 (−3.99**, −0.18)^**^	0.04 (0.85)
T3	−1.2 (−3.81, 1.42)	−2.35 (−5.04, 0.34)^*^	1.02 (0.06)^*^	−0.23 (−0.46, 0.01)^*^	**−0.31 (−0.55**, −0.07)^**^	0.08 (0.09)^*^	−1.32 (−3.37, 0.73)	−1.98 (−4.08, 0.12)^*^	0.6 (0.11)
PA visit 1	0.61 (−2.67, 3.88)	0.09 (−3.08, 3.26)	0.54 (0.18)	0.1 (−0.23, 0.43)	0.07 (−0.25, 0.39)	0.03 (0.26)	−0.31 (−3.11, 2.49)	−0.57 (−3.32, 2.18)	0.27 (0.28)
MEHP									
T1	−1.95 (−4.46, 0.56)	−2.1 (−4.56, 0.36)	0.29 (0.4)	−0.13 (−0.38, 0.12)	−0.14 (−0.38, 0.11)	0.02 (0.42)	−1.57 (−3.33, 0.2)^*^	−1.73 (−3.49, 0.03)^*^	0.18 (0.41)
T2	−2.07 (−4.97, 0.83)	−1.57 (−4.29, 1.15)	−0.52 (0.25)	−0.19 (−0.5, 0.13)	−0.15 (−0.45, 0.14)	−0.03 (0.32)	−1.72 (−4.54, 1.1)	−1.48 (−4.21, 1.24)	−0.28 (0.34)
T3	−1.35 (−4.39, 1.7)	−0.69 (−3.91, 2.53)	−0.73 (0.13)	−0.11 (−0.36, 0.14)	−0.07 (−0.33, 0.19)	−0.05 (0.2)	−0.4 (−2.6, 1.79)	−0.07 (−2.29, 2.15)	−0.41 (0.2)
PA visit 1	−0.77 (−4.14, 2.61)	−0.29 (−3.58, 3.01)	−0.47 (0.31)	−0.03 (−0.35, 0.29)	0 (−0.32, 0.31)	−0.03 (0.35)	−0.6 (−3.42, 2.21)	−0.37 (−3.14, 2.4)	−0.22 (0.38)
***H19*** **CpG #1 AS MEDIATOR**									
MBP									
T1	1.88 (−0.42, 4.18)	1.54 (−0.85, 3.93)	0.33 (0.22)	0.25 (0.03, 0.46)^**^	0.22 (0, 0.44)^*^	0.02 (0.37)	1.72 (−0.09, 3.53)^*^	1.48 (−0.39, 3.35)	0.17 (0.34)
T2	−1.45 (−3.67, 0.77)	−1.1 (−3.41, 1.2)	−0.27 (0.35)	−0.12 (−0.32, 0.08)	−0.09 (−0.29, 0.11)	−0.01 (0.44)	−1.2 (−2.94, 0.53)	−0.99 (−2.73, 0.76)	−0.12 (0.47)
T3	0.05 (−2.23, 2.32)	0.24 (−2.2, 2.69)	−0.17 (0.58)	−0.06 (−0.26, 0.14)	−0.04 (−0.24, 0.16)	−0.01 (0.59)	−0.36 (−2.09, 1.36)	−0.22 (−1.96, 1.52)	−0.08 (0.6)
PA visit 1	−2.28 (−4.86, 0.29)^*^	−2.36 (−4.97, 0.25)^*^	0.19 (0.51)	−0.21 (−0.46, 0.03)^*^	−0.21 (−0.47, 0.04)	0.01 (0.54)	−1.63 (−3.65, 0.4)	−1.67 (−3.74, 0.4)	0.07 (0.56)
MiBP									
T1	3.41 (1.5, 5.31)^***^	2.98 (0.84, 5.12)^**^	0.38 (0.21)	0.28 (0.12, 0.45)^***^	0.26 (0.08, 0.44)^***^	0.02 (0.48)	2.33 (0.86, 3.8)^***^	2.13 (0.47, 3.79)^**^	0.19 (0.42)
T2	1.46 (−0.76, 3.68)	0.95 (−1.57, 3.48)	0.49 (0.17)	0.03 (−0.19, 0.24)	−0.01 (−0.25, 0.23)	0.03 (0.31)	0.55 (−1.61, 2.72)	0.39 (−2.02, 2.8)	0.23 (0.39)
T3	2.17 (−1.23, 5.56)	1.85 (−1.74, 5.45)	0.37 (0.35)	0.1 (−0.2, 0.4)	0.08 (−0.23, 0.4)	0.02 (0.41)	1.55 (−1.14, 4.23)	1.53 (−1.26, 4.32)	0.17 (0.43)
PA visit 1	−0.38 (−3.23, 2.47)	−0.23 (−3.17, 2.7)	0.16 (0.64)	−0.1 (−0.39, 0.2)	−0.08 (−0.38, 0.22)	0.01 (0.66)	−1.15 (−3.29, 0.99)	−1.13 (−3.33, 1.08)	0.06 (0.67)
MBzP									
T1	−0.1 (−2.21, 2.00)	−0.65 (−2.75, 1.45)	0.44 (0.18)	0.04 (−0.18, 0.25)	0 (−0.21, 0.22)	0.03 (0.3)	0.03 (−1.7, 1.76)	−0.24 (−2.01, 1.53)	0.24 (0.28)
T2	**−2.53 (−4.78**, −0.28)^**^	**−2.73 (−4.82**, −0.65)^**^	0.08 (0.77)	−0.22 (−0.46, 0.03)^*^	−0.23 (−0.46, 0.01)^*^	0.00 (0.77)	−2.01 (−4.07, 0.05)^*^	**−2.09 (−4.06**, −0.12)^**^	0.04 (0.77)
T3	−1.2 (−3.81, 1.42)	−2.23 (−4.91, 0.45)	0.9 (0.07)^*^	−0.23 (−0.46, 0.01)^*^	**−0.29 (−0.53**, −0.05)^**^	0.06 (0.12)	−1.32 (−3.37, 0.73)	−1.84 (−3.96, 0.27)^*^	0.47 (0.17)
PA visit 1	0.61 (−2.67, 3.88)	0.26 (−2.9, 3.41)	0.37 (0.28)	0.1 (−0.23, 0.43)	0.08 (−0.24, 0.4)	0.02 (0.39)	−0.31 (−3.11, 2.49)	−0.45 (−3.21, 2.31)	0.15 (0.43)
MEHP									
T1	−1.95 (−4.46, 0.56)	−2.04 (−4.53, 0.44)	0.23 (0.45)	−0.13 (−0.38, 0.12)	−0.13 (−0.38, 0.12)	0.01 (0.48)	−1.57 (−3.33, 0.2)^*^	−1.69 (−3.46, 0.09)^*^	0.13 (0.48)
T2	−2.07 (−4.97, 0.83)	−1.75 (−4.5, 1.00)	−0.34 (0.34)	−0.19 (−0.5, 0.13)	−0.17 (−0.47, 0.13)	−0.02 (0.45)	−1.72 (−4.54, 1.1)	−1.61 (−4.38, 1.16)	−0.14 (0.48)
T3	−1.35 (−4.39, 1.7)	−0.76 (−3.94, 2.41)	−0.66 (0.16)	−0.11 (−0.36, 0.14)	−0.09 (−0.34, 0.17)	−0.04 (0.27)	−0.4 (−2.6, 1.79)	−0.17 (−2.37, 2.04)	−0.32 (0.28)
PA visit 1	−0.77 (−4.14, 2.61)	−0.23 (−3.5, 3.04)	−0.53 (0.24)	−0.03 (−0.35, 0.29)	0 (−0.32, 0.31)	−0.03 (0.35)	−0.6 (−3.42, 2.21)	−0.4 (−3.16, 2.37)	−0.2 (0.43)

*Effect estimates (95% CI) are reported in the table*.

* p < 0.1,

** ***p <0.05***,

*** ***p <0.01***.

*Phthalate concentrations (μg/L) were natural log transformed. Total effects represent the effect estimate of the phthalate concentrations on measures of adiposity from a generalized estimating equation relating repeated measures of adiposity to phthalates leaving the potential mediator (DNA methylation at H19 CpG sites 1 or 4) out of the model and adjusting for specific gravity, maternal education, and age. Direct effects represent the effect estimate of phthalate concentration in the same model but with DNA methylation also included. The indirect effect is the product of the effect of the exposure on the mediator and the effect of the mediator on the outcome. The Sobel test was used to test significance of the mediation pathway*.

**Table 7:** Mediation analysis among males.

	**Skinfold thickness (mm)**			**B MI for age z–s core**			**Waist circumference (cm)**		
**Exposure**	**Total effect**	**Direct effect**	**Indirect effect (Sobel *p*–value)**	**Total effect**	**Direct effect**	**Indirect effect (Sobel *p*–value)**	**Total effect**	**Direct effect**	**Indirect effect (Sobel *p*–value)**
***HSD11B2*** **CpG #1 AS MEDIATOR**									
MEHP									
T1	2.67 (−0.37, 5.71)^*^	3.12 (−0.09, 6.33)^*^	−0.48 (0.27)	0.28 (0.002, 0.57)^**^	0.31 (0.01, 0.60)^**^	−0.02 (0.40)	1.86 (−0.61, 4.33)	2.25 (−0.3, 4.8)^*^	−0.39 (0.26)
T2	0.63 (−2.42, 3.67)	0.9 (−2.13, 3.93)	−0.24 (0.55)	0.23 (−0.08, 0.55)	0.25 (−0.06, 0.56)	−0.01 (0.58)	1.41 (−0.91, 3.74)	1.61 (−0.67, 3.9)	−0.19 (0.55)
T3	−0.63 (−3.63, 2.37)	0.3 (−2.34, 2.94)	−0.89 (0.11)	0.09 (−0.18, 0.37)	0.18 (−0.08, 0.43)	−0.06 (0.21)	−0.34 (−2.57, 1.89)	0.37 (−1.67, 2.41)	−0.68 (0.12)
PA visit 1	−1.90 (−3.91, 0.11)^*^	−1.87 (−3.76, 0.02)^*^	−0.24 (0.37)	−0.15 (−0.36, 0.05)	−0.16 (−0.36, 0.04)	−0.01 (0.68)	−1.33 (−2.9, 0.24)	−1.31 (−2.86, 0.23)	−0.11 (0.47)
MBzP									
T1	1.91 (−0.3, 4.12)^*^	1.71 (−0.41, 3.83)	0.23 (0.37)	0.19 (−0.001, 0.39)^*^	0.18 (−0.01, 0.38)^*^	0.01 (0.59)	1.55 (−0.13, 3.22)^*^	1.38 (−0.22, 2.99)^*^	0.19 (0.36)
T2	2.00 (−0.43, 4.43)	2.06 (−0.24, 4.37)^*^	0.07 (0.83)	0.25 (0.01, 0.49)^**^	0.26 (0.02, 0.50)^**^	0.00 (0.83)	2.11 (0.27, 3.95)^**^	2.13 (0.37, 3.88)^**^	0.05 (0.83)
T3	−0.49 (−2.93, 1.94)	−1.35 (−3.8, 1.1)	0.84 (0.12)	−0.12 (−0.33, 0.09)	−0.17 (−0.40, 0.07)	0.05 (0.25)	−0.35 (−2.24, 1.55)	−0.98 (−2.98, 1.02)	0.63 (0.14)
PA visit 1	**−2.43 (−4.69**, −0.17)^**^	−2.29 (−4.58, −0.01)^*^	−0.11 (0.73)	−0.17 (−0.41, 0.07)	−0.17 (−0.42, 0.08)	0.00 (0.77)	**−1.91 (−3.64**, −0.19)**	**−1.86 (−3.6**, −0.11)^**^	−0.05 (0.73)
***HSD11B2*** **CpG #2 AS MEDIATOR**									
MEHP									
T1	2.67 (−0.37, 5.71)^*^	3.02 (−0.1, 6.14)^*^	−0.37 (0.34)	0.28 (0.002, 0.57)^**^	0.32 (0.04, 0.6)^**^	−0.04 (0.33)	1.86 (−0.61, 4.33)	2.19 (−0.2, 4.59)^*^	−0.33 (0.33)
T2	0.63 (−2.42, 3.67)	0.83 (−2.32, 3.98)	−0.17 (0.69)	0.23 (−0.08, 0.55)	0.26 (−0.06, 0.57)	−0.02 (0.69)	1.41 (−0.91, 3.74)	1.58 (−0.76, 3.91)	−0.15 (0.69)
T3	−0.63 (−3.63, 2.37)	−0.51 (−3.4, 2.38)	−0.07 (0.80)	0.09 (−0.18, 0.37)	0.12 (−0.15, 0.39)	−0.01 (0.80)	−0.34 (−2.57, 1.89)	−0.25 (−2.42, 1.92)	−0.06 (0.80)
PA visit 1	−1.90 (−3.91, 0.11)^*^	−1.52 (−3.34, 0.31)	−0.64 (0.26)	−0.15 (−0.36, 0.05)	−0.11 (−0.33, 0.1)	−0.06 (0.38)	−1.33 (−2.9, 0.24)	−1.01 (−2.59, 0.57)	−0.43 (0.34)
MBzP									
T1	1.91 (−0.30, 4.12)^*^	2.09 (−0.16, 4.33)^*^	−0.16 (0.55)	0.19 (−0.001, 0.39)^*^	0.21 (0.01, 0.40)^**^	−0.02 (0.54)	1.55 (−0.13, 3.22)^*^	1.71 (0.06, 3.37)^**^	−0.15 (0.54)
T2	2.00 (−0.43, 4.43)	1.95 (−0.38, 4.27)	0.19 (0.54)	0.25 (0.01, 0.49)^**^	0.25 (0.01, 0.48)^**^	0.02 (0.54)	2.11 (0.27, 3.95)^**^	2.02 (0.27, 3.76)^**^	0.16 (0.53)
T3	−0.49 (−2.93, 1.94)	−0.6 (−2.95, 1.74)	0.08 (0.68)	−0.12 (−0.33, 0.09)	−0.12 (−0.32, 0.08)	0.01 (0.68)	−0.35 (−2.24, 1.55)	−0.42 (−2.23, 1.39)	0.07 (0.68)
PA visit 1	**−2.43 (−4.69**, −0.17)^**^	−2.04 (−4.23, 0.15)^*^	−0.39 (0.25)	−0.17 (−0.41, 0.07)	−0.14 (−0.39, 0.11)	−0.03 (0.32)	**−1.91 (−3.64**, −0.19)^**^	−1.65 (−3.35, 0.04)^*^	−0.26 (0.30)

*Effect estimates (95% CI) are reported in the table*.

* p < 0.1,

** **p < 0.05.**

*Phthalate concentrations (μg/L) were natural log transformed. Total effects represent the effect estimate of the phthalate concentrations on measures of adiposity from a generalized estimating equation relating repeated measures of adiposity to phthalates leaving the potential mediator (DNA methylation at HSD11B2 CpG sites 1 or 2) out of the model and adjusting for specific gravity, maternal education, and age. Direct effects represent the effect estimate of phthalate concentration in the same model but with DNA methylation also included. The indirect effect is the product of the effect of the exposure on the mediator and the effect of the mediator on the outcome. The Sobel test was used to test significance of the mediation pathway*.

**Table 8:** Sample size requirement for significant Sobel test result given varying strengths of effect in a longitudinal model adjusted for covariates.

		**Magnitude of standardized effect estimate of mediator on outcome, adjusted for exposure and covariates**		
		**Weak (0.10)**	**Medium (0.15)**	**Strong (0.20)**
Magnitude of standardized effect of exposure on mediator, adjusted for covariates	Weak (0.04)	873	819	802
	Medium (0.10)	222	167	150
	Strong (0.29)	121	61	43

**Figure 1:**
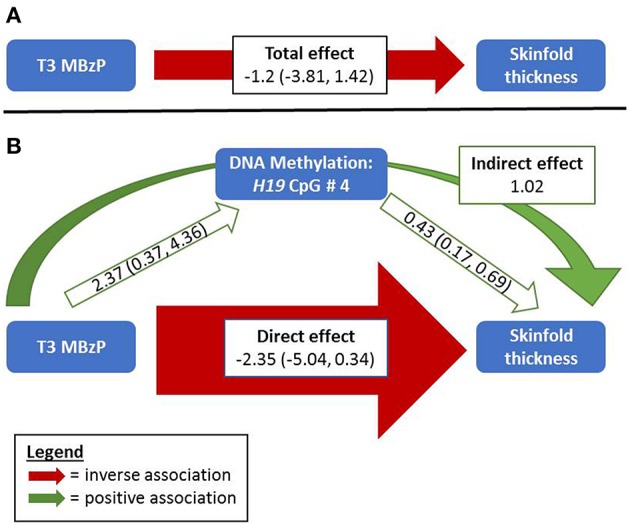
Relationships between MBzP, DNA Methylation, and Skinfold Thickness among Girls. **(A)** Without considering DNA methylation, third trimester MBzP exposure is negatively associated with repeat measures of skinfold thickness among girls measured at two time points in late childhood/adolescence. **(B)** Including DNA methylation of *H19* as a potential mediator in the model reveals a stronger negative direct effect of MBzP on skinfold thickness that was masked by the positive relationships between *H19* DNA methylation with both MBzP and adiposity. The Sobel test for non-zero mediation in this example nears statistical significance (*p* = 0.06). While this example shows effect estimates for *H19* CpG 4 and skinfold thickness, similar relationships are observed with *H19* CpG sites 1 and 4 with three adiposity measures among girls (Supplemental Table 1). Width of the arrows indicate relative magnitude of association, although they are not to scale. The total and direct effects reported here are estimated parameters from models of the outcome without and with the mediator, respectively. The indirect effect is from a non-parametric calculation (the product of the effects of the exposure on the mediator and of the mediator on the outcome).

In girls, the Sobel test for non-zero mediation neared significance for *H19* CpG sites 1 and 4 in the association between T3 MBzP and skinfold thickness (*p* = 0.07 and 0.06, respectively); there was also evidence for *H19* site 4 mediating the relationship with MBzP and BMI-for-age z-score (*p* = 0.09). Figure 1 illustrates this potential attenuating relationship by which MBzP exposure increases *H19* DNA methylation which is in turn associated with increased adiposity. However, the direct effect of MBzP on adiposity is negative. In sum, T3 MBzP is inversely associated with skinfold thickness through the direct pathway and is positively associated with skinfold thickness through the mediation pathway with the result of an overall attenuating effect of *H19* methylation on the inverse relationship between MBzP and skinfold thickness. Instances in which the direct and indirect effects have differing directions of association have previously been described as “inconsistent mediation” (32), and may reflect situations where the mediator, in this case DNA methylation, is protecting against the effect of the exposure.

Among girls, an example of mediation in the expected direction (e.g., the exposure is working through the mediator to influence an outcome), can be seen with *H19* methylation at CpG sites 1 and 4 on the relationship between T2 MiBP and adiposity outcomes. In all cases, including DNA methylation in the model attenuates the direct effect of T2 MiBP on adiposity by at least 30%, though the Sobel tests for non-zero mediation are not significant (*p*-values 0.11 to 0.39).

In boys, no statistical tests for non-zero mediation reached significance (Sobel *p* > 0.1). Though similar to the girls analysis, there were mediating pairs that displayed estimates indicative of classic mediation (e.g., MBzP from PA visit 1 with *HSD11B2* CpG #2 on skinfold thickness and waist circumference) that merit further study in a largerH sample size.

## Discussion

Our results show that phthalate exposures during pregnancy and in early adolescence have sex-specific associations with DNA methylation and sex- and exposure timing-specific associations with repeat measures of adolescent adiposity. Even though the analysis was underpowered to detect mediation pathways with small to medium effect sizes, there was suggestive evidence for DNA methylation as a mediator between phthalate exposure and adiposity (Sobel *p* > 0.05). For example among girls, the positive associations between T1 or T2 MiBP and adiposity may be explained in part by the indirect effect of increased *H19* methylation. On the other hand, accounting for DNA methylation may reveal stronger direct relationships between exposures and outcomes in cases when epigenetic change may be protecting against the effects of exposure. Among girls, controlling for *H19* methylation revealed a stronger inverse association between T3 MBzP and skinfold thickness (Figure 1) or BMI-for-age (Table 6). This highlights the importance of considering epigenetics as a mediator not only in the traditional sense but also as a biological buffer that may influence susceptibility to effects from toxicant exposures.

This study expands upon previous ELEMENT research reporting associations between phthalates from T3 and PA visit 1 with adiposity at PA visit 1 (10) to include exposure measures in the first two trimesters and repeated anthropometry measures ~3 years later as children progress through puberty. There were consistent positive associations between T1 metabolites of the low-molecular weight parent phthalate, DBP (MBP and MIBP), with adiposity among girls. The prenatal association is similar to the results from a cohort of Mexican American children which found an increased odds for overweight and obesity at age 12 years with a doubling in prenatal DBP, though that association was only observed among boys (8). A study conducted by Deierlein et al. found that the sum of low molecular weight phthalates measured at ages 6–8 years was positively associated with repeat measures of BMI and waist circumference several years later among American girls (33). Collectively, these studies point to the importance of timing of outcome and exposure to DBP on adiposity. The importance of timing was previously demonstrated for repeat measures of phthalates through age 8 years and body fat at 8 years in the Health Outcomes and Measures of the Environment (HOME) study. The HOME study reported different directions of associations for some phthalates with body fat in the same children based on whether exposure was assessed in pregnancy, early, or mid-childhood (9).

Among girls, the high molecular weight phthalate metabolite MBzP (T2) was associated with decreased adiposity. A similar relationship was reported for gestational MBzP and decreased body fat at 8 years of age among all children of the HOME study that was near significant (9). However, in the ELEMENT cohort T2 MBzP was associated with increased adiposity measures among boys yet decreased adiposity if the exposure occurred in childhood. These results show the importance of stratifying by sex when assessing the effects of phthalates and of considering multiple exposures during development. While the ELEMENT study along with others gives evidence for the relationship between phthalate exposures in pregnancy or childhood and adiposity, inconsistencies exist across studies (5, 8, 9, 12, 33–35). Inconsistencies may be due to differences in timing of exposure and outcome assessments (e.g., pre-puberty vs. post-puberty), statistical analysis methods (e.g., individual vs. summed phthalates, sex-stratified vs. all children), and population including underlying susceptibility factors (e.g., genetics, epigenetics, other confounders).

Biological mechanisms such as epigenetic alteration may underlie susceptibility to effects on adiposity from phthalate exposures at key developmental windows. While evidence exists for the influence of phthalates, especially gestational exposure, on the epigenome (20–22, 36), this is the first study to examine DNA methylation as a mediator between exposure and adiposity. The *H19* imprint control region (ICR) and promoter of *HSD11B2* were interrogated due to the functions of these genes in growth regulation as well as previous associations in the ELEMENT cohort between phthalate exposures and DNA methylation at these genes (20). We previously examined relationships between PA visit 1 DNA methylation at four candidate regions and T3 or PA visit 1 exposure biomarkers for phthalates, bisphenol A and lead. Among all children, we reported increased *H19* methylation with T3 MIBP and MBzP and increased *HSD11B2* methylation with concurrent MEHP (20). Of note in the new analyses reported here that include additional exposure measures in T1 and T2, the association between MIBP and *H19* methylation appears to be stronger when the exposure is assessed in T1 among girls (Supplemental Table 1). First trimester is a vulnerable period since the epigenome is reprogrammed in early gestation, and changes from environmental perturbation at this time can be propagated across germ layers (37). In a sample of 17-year old children from Australia, blood leukocyte DNA methylation at the same *H19* ICR was associated with skinfold thickness in a cross-sectional analysis (23). We observed a similar positive association among girls between *H19* methylation and repeat measures of skinfold thickness in this study. Among boys, *HSD11B2* methylation was inversely related to repeat measures of skinfold thickness, BMI z-score, and waist circumference, though only at the 90% confidence level. The longitudinal analysis of these associations suggests that DNA methylation of growth related genes has a persistent association with adiposity throughout adolescence that is sex-specific.

While several studies have examined mediating relationships between phthalate exposure, DNA methylation, and various health outcomes, previous studies have been cross-sectional, focused on outcomes other than adiposity, and/or focused on outcomes in early life. For example, there was cross-sectional evidence for blood leukocyte DNA methylation at *TNF-alpha* mediating the relationship between MEHP and asthma among children from three study populations (16). In another study, repetitive element cord blood DNA methylation was not found to be a mediator in the relationship between prenatal phthalate exposures and birth outcomes (38). Our analysis did not reveal any statistically significant mediating pathways from phthalate exposure to DNA methylation to adiposity in boys or girls. However, our sex-stratified analysis was underpowered to detect mediation except in the case of large effect sizes (Table 8), and environmentally-induced epigenetic changes are typically small (39). Though not statistically significant, results from the mediation analysis suggest that DNA methylation could serve as a mediator of toxicant effects in some instances or as a protective mechanism influencing susceptibility to effects in other cases. For example, when controlling for *H19* methylation, the association of T1 or T2 MiBP with adiposity outcomes among girls are attenuated, and there is a positive indirect effect on adiposity through this potential mediator. On the other hand, controlling for *H19* methylation revealed a stronger direct association between T3 MBzP and skinfold thickness and BMI z-score among girls (Sobel *p*-values 0.06 and 0.09). These two examples present a framework for future research examining epigenetic mediation of exposure-outcome relationships that should consider not only classical mediation (i.e., exposure working through the mediator) but also consider epigenetics as a buffer that reduces the total effect from exposures.

Strengths of this study include the longitudinal design with outcome measurements at two times in peri-adolescence as well as three exposure measures during pregnancy allowing assessment of different windows of vulnerability during pre- and post-natal development. Additionally, including multiple outcome measures allowed for a validity assessment of adiposity, with directions of association remaining fairly constant across each adiposity proxy. The selection of genes was hypothesis driven based on gene function and previously reported associations with exposure. While a strength, this was also a limitation since additional genes that influence growth and adiposity may be environmentally-responsive and were not examined here. Validity of spot urine concentrations as measures of average exposure is limited due to the short biological half-life of phthalates (40). However, because exposure to phthalates often come from ubiquitous and/or chronic sources, exposure levels are thought to be relatively stable (41). Blood leukocyte DNA represents a variety of cell types, yet we cannot adjust for the cell type proportions as a differential was not obtained at the time of blood sample collection. Statistical power in this study is limited for detecting low to medium mediation effect sizes due to the stratified sample size. Thus, mediation by *H19* and *HSD11B2* methylation, or other genes not included here, should be explored in future studies on phthalates' effects. Further, due to limited statistical power, our analysis did not test for interactions between exposures and mediators. With the modeling strategy we used, the direct effect is equivalent to a controlled direct effect in the absence of exposure-mediator interaction. If such an interaction exists, the controlled direct effect would need to be computed based on varying levels of the mediator.

In summary, timing and sex-specific associations between phthalates and measures of adiposity in Mexican children assessed twice between ages 8 and 17 years were observed. DNA methylation at growth-regulating genes was also associated with exposures and outcomes. While our mediation analysis was underpowered, epigenetic regulation of these and other related genes may represent avenues by which exposures can exert their effects or by which children can be protected from effects. Since epigenetic profiles vary by race/ethnicity, sex, and age, it is important for future research to consider epigenetics as a susceptibility factor influencing exposure-outcome relationships.

## Ethics Statement

Mothers received detailed information of study procedures and signed a letter of informed consent at initial recruitment and at follow up in accordance with the Declaration of Helsinki. Children provided assent in written or verbal forms when age-appropriate for follow-up visits. Research protocols were approved by the Ethics and Research Committees of participating institutions in Mexico and the USA including at the University of Michigan.

## Author Contributions

AB, KP, JG, DD, MT-R, and JM contributed to conception and design of the study. AB performed the statistical analysis and wrote the first draft of the manuscript. JG provided oversight in the analysis and writing processes. MT-R, AM-G, KP, DD, JM, BS, and JG were responsible for obtaining data from the cohort and following the cohort. All authors contributed to the manuscript and approved the submission.

### Conflict of Interest Statement

The authors declare that the research was conducted in the absence of any commercial or financial relationships that could be construed as a potential conflict of interest.
